# Reasons for Nonattendance across the Hepatitis C Disease Course

**DOI:** 10.1155/2013/579529

**Published:** 2013-09-11

**Authors:** Gail Butt, Liza McGuinness, Terri Buller-Taylor, Sandi Mitchell

**Affiliations:** ^1^UBC/BCCDC, 655 West 12th Avenue, Vancouver, BC, Canada V5Z 4R4; ^2^BC Centre for Disease Control, 655 West 12th Avenue, Vancouver, BC, Canada V5Z 4R4

## Abstract

This descriptive qualitative study examined the patient, provider, and institutional factors contributing to nonattendance for hepatitis C (HCV) care throughout the disease course. Eighty-four patients and health and social care providers were interviewed. Thematic analysis of the data yielded 6 interrelated nonattendance themes: self-protection, determining the benefits, competing priorities, knowledge gaps, access to services, and restrictive policies. Factors within the themes varied with the disease course, type of provider/service, and patient context. Nonattendance could span months to years and most frequently began at diagnosis where providers either advised that followup was not necessary or did not recommend any followup. The way services were organized (low barrier access) and delivered (nonjudgmental approach) and higher HCV knowledge levels of patients and providers encouraged attendance. This is the first study to explore the reasons for nonattendance for HCV care throughout the disease course and validate them from multiple perspectives. There are missed opportunities for providers to encourage attendance throughout the disease course beginning at diagnosis. Interventions required include development of integrated health and social service delivery models; mechanisms to improve knowledge dissemination of the disease, its management, and treatment; and implementation of standardized followup protocols for liver disease monitoring in primary care.

## 1. Introduction 

We report the findings from qualitative research to ascertain the patient, provider, and institutional factors that contribute to nonattendance for care for hepatitis C virus (HCV) infection at various points along the disease course. 

 HCV is a chronic infectious disease, spread through blood to blood contact that affects 170 million worldwide, including approximately 250,000 Canadians [1]. Because HCV has a heterogeneous presentation and a slow, unpredictable course of liver inflammation that spans decades, patients require long-term monitoring for signs of progressive liver disease and associated issues such as cirrhosis, end-stage liver disease, liver cancer, HIV coinfection, fatty liver disease, and alcohol overuse [2–8]. 

Patients receive diagnostic and most followup services in primary care due to the large number affected and the relative shortage of specialists [9]. Specialists provide complex disease management for those with advanced disease including the provision of a 24- to 48-week course of antiviral therapy for those eligible, consenting, and able to tolerate it. Treatment uptake has been historically low, even though antiviral therapy can permanently arrest viral replication (initially in 10% but presently in 70% of cases) [10], improve quality of life and liver function, decrease the likelihood of developing liver cancer, and improve survival [11, 12]. 

 Disease management, whether in primary or specialist settings, can assist patients to manage symptoms and prevent liver disease acceleration [13]. Despite these benefits, patient nonattendance rates range from 28 to 80% [14–16], and little is known about the reasons for this. Nonattendance results in wasted clinician time and health care resources, but a more critical issue is the delay in presentation and lack of monitoring and management that can predispose the patient to complications unnecessarily. 

 Gaps in HCV care have been noted to result from patient and provider actions. Many patients begin HCV testing but do not return to obtain their results or complete the diagnostic testing process [16–18]. Other gaps occur when patients with a history of nonattendance in primary care are not offered a specialist referral by their provider [14, 19, 20]. Of those who are referred, many fail to attend the specialist appointment. A small study that looked at reasons why patients referred to a specialist clinic who did not attend found fear of the unknown precipitated nonattendance, for example, fear of being asked to undergo a liver biopsy, or being told that they had advanced disease or were going to die [21]. The research did not include factors such as whether patient preparation at the primary care level affects nonattendance for specialist assessment. For those under specialist care, studies have identified patient factors specific to the decision to decline or defer antiviral therapy which include concern over side effects, inconvenient time to start, belief that the disease is not serious enough to need intervention, disbelief in treatment efficacy, ongoing drug or alcohol use, and chaotic lifestyles [22–26].

Nonattendance has been noted as an outcome of HCV stigma from health care provider and institutional policies/practices. For example, policies that restrict eligibility for HCV treatment and agencies that give preference to patients with certain characteristics are viewed by patients as stigmatizing and result in nonattendance [27]. HCV stigma is thought to be rooted in a lack of knowledge of HCV and its association with injection drug use [27, 28]. 

HCV nonattendance research, although sparse, is consistent with findings from other chronic diseases which indicate that the factors affecting nonattendance are complex and involve the patient, the provider, and the health care system [29]. As a first step towards enhancing our understanding of the HCV nonattendance issues, we report the findings of descriptive research to identify, analyse, and describe patient, provider, and institutional factors contributing to nonattendance throughout the disease course [30]. The purpose of this paper is to describe the breadth of the nonattendance issue and identify areas for action. 

## 2. Methods 

A descriptive design was used to answer the research question, what are the reasons individuals with HCV do not attend for HCV care? Nonattendance was defined as instances when patients did not attend appointments and when patients delayed or deferred care. To extend our understanding of the perceived contributing factors, patients and HCV providers from multiple contexts were engaged in the research. 

### 2.1. Ethics

Ethics approval was granted from two required review boards. Prior to interviews, written and oral information was given to all participants to ensure informed consent. To maximize confidentiality and accessibility, patient participants were interviewed by phone on a toll-free line with the exception of four patients who requested in-person interviews. To facilitate participation by patients without phones, community support agencies provided a private space and telephone. Data confidentiality was maintained using codes in place of personal identifiers and restricting transcript and data access to the research team. Participants received a $40 honorarium for their time and incidental costs. 

### 2.2. Data Collection, Management, and Analysis

Purposeful sampling techniques were used to obtain a varied sample of “patient” and “provider” participants [31]. Patients were recruited through advertising by staff in health promotion, harm reduction, health, and social service programs and through snowballing, where participants recruit others directly or through postings on their blogs or online groups. Providers who were experienced in hepatitis C care were recruited through advertisements circulated by the research team and their provider networks. 

Patient interviews lasted 45 to 90 minutes. Three trained interviewers used standard questions to collect demographic data followed by broad open-ended queries that led to more individual-focused questions to elicit the patient's experiences with HCV care and specifically instances of nonattendance. An interview guide, based on our previous work and input from an expert advisory committee of patients and providers, was used to facilitate exploration and enrich the data [32]. Ninety-minute interviews were conducted in-person with groups of 6 to 9 providers by the principle investigator (PI) (GB) for consistency [31]. All interviews were audio-recorded, transcribed verbatim, and thematically analyzed. Analysis occurred concurrent with data collection to facilitate identification of new questions and areas for further exploration or clarification [30]. 

Data were managed through NVivo 9 software which enabled separation of patient and provider data, data interrogation, refinement of the coding structure, and themes. The first five transcripts were read, reread, and manually coded by the PI (GB). The other team members (LM, SM) subsequently read, reread, and coded the same transcripts. The team then produced an inclusive list of codes through consensus. The coding structure was refined at weekly team meetings. Notes were kept to record thoughts, interpretations, questions, and decisions, about the data and its interpretation. Thematic analysis allowed for the data to be interpreted and organized into themes through inductive coding and systematic classification. For example, codes that described the issues considered by patients when deciding whether to attend for care were grouped under the theme “determining the benefits.” Recruitment ceased shortly after no new codes or themes were identified. 

### 2.3. Sample Characteristics

Participants included 55 patients with self-reported HCV and 29 HCV health and social care providers from 5 provinces: Nova Scotia, Quebec, Ontario, Manitoba, and British Columbia. Patient demographic data in Table 1 reveals that although patients tended to be over 40 years (73%) and well educated (69% had 12 or more years of formal education), most required income support (76%), a need they attributed to their poor health status. Patients reported difficulty determining which symptoms to ascribe to HCV as they had a mean of 4 other coexisting conditions (range 1 to 9) including arthritis, diabetes, digestive problems, chronic pain, addictions, anxiety, and depression. Their mean number of medications was 3 (range 0 to 15), with methadone, antidepressants, sleeping pills, analgesics, and antihypertensives being the most frequently reported. 

Years since HCV diagnosis ranged from 1 to more than 20. Four (7%) had cleared the virus spontaneously. Of the 22 (40%) patients who had experienced antiviral therapy, 13 (59%) attained viral clearance, while 2 were currently on therapy. 

The 29 providers represented a range of agencies including public health (8), prison health (3), street outreach (1), community clinics (4), community support agencies (7), and specialist clinics (6). Mean years experience working with HCV patients was 8.5 (range <1 to 15+ years; *n* = 20/29). 

## 3. Results 

Patient and provider data, although analysed separately, revealed the same six themes, evidencing congruence between patient perspectives and that of providers that specialize in working with HCV patients. The six themes were self-protection; determining the benefits; competing priorities; knowledge gaps; access to services; and restrictive policies. The first four themes center around the patient and provider at the individual and interpersonal levels, while the final two themes align with the institutional (system) level. The results are presented using examples where it enhances the explanation or description. 

### 3.1. Self-Protection

This theme represents nonattendance as a self-protective response precipitated by the perception of being negatively judged or treated differently by providers on patient disclosure of HCV infection. Providers echoed the powerful and pervasive patient accounts. The participants perceived that negative provider behaviours were closely linked to a lack of HCV knowledge and the association of HCV with injection drug use. Patients described strong emotional responses to negative experiences in primary care, emergency care, and to a lesser extent specialty care. The emotional response and the need to prevent further negative events were so strong that many patients not only avoided disclosing their status to potential sources of support but also described periods of up to several years in which they did not attend care or changed providers. 

In primary care settings, patients relayed that, beginning at HCV diagnosis, they felt that they were unfairly judged or treated as a “drug user.” A few patients described delaying diagnostic testing to keep their HCV status a secret in order to protect themselves from anticipated negative experiences. Patients described many instances in which they felt obliged to disclose their HCV status resulting in a “go to the back of the line” experience or providers indicating, implicitly or explicitly, that they were “not worthy of care.” Implications of disclosure were magnified in patient accounts from small, rural, and remote communities as confidentiality is difficult to maintain and there are few choices of where to receive services. Other difficulties arose if providers occupied additional roles such as cultural or religious leader. In one example, the patient's physician was also a religious leader, so the patient felt conflicted as disclosure of the behaviour that led to HCV infection was in opposition to religious norms. The possibility that diagnosis or disclosure could lead to service termination by the practitioner created a patient dilemma. 

Many participants described care in emergency departments as “rude” and “judgmental.” Patients described being treated “like a dog” or “like dirt.” In some cases they were accused of drug seeking and/or discharged without assessment for their presenting problem. Many participants pointed out that women found emergency room experiences particularly difficult and that they refused to seek emergency services. One participant related that his partner delayed attending for so long she died within hours of entering hospital:

*cuz they treated her like she was a dog … she'd go out and get dope, you know, four blocks from the hospital but she wouldn't go to the hospital, you know, and she basically they uh, one day the ambulance picked her up, took her to the hospital and she was dead by midnight. *

In contrast, walk-in clinics were frequented, and when explored further, it was perceived that the way clinics are run, for example, no appointments were required and approachable staff created a comfortable atmosphere. Patients explained that walk-in clinic staff had more experience working with people with HCV and were more knowledgeable about the disease. 

Although disclosure avoidance was rarely mentioned in relation to specialist care, some patients expressed discomfort with sitting in waiting rooms because of judgments they perceived other patients might make about them. Providers recounted that patients delay or defer treatment because they fear that someone they know will guess their diagnosis if they are seen attending hepatitis clinics or have visible symptoms during treatment. One provider recounted a particular client who struggled with the decision to start treatment: “she did not want to go to be treated, because she said everybody knows me, I'm well known in this city … if I come here to visit you and to see you they'll know what I'm here for.” 

Patients who received intensive phone support during treatment were appreciative as it was easily accessed and more anonymous than an office visit. Providers also noted that flexibility and willingness to provide phone support could act as an attendance facilitator. Some questioned the effectiveness of strict requirements for frequent in-person visits during treatment citing clients' expressions of difficulty, especially when they were ill from the treatment effects. 

Patients and providers expressed concern that an unintended consequence of disclosure avoidance is that few patients speak out about the issues they face or lobby for the type of services they need to improve their care. Examples of needed support services included peer and group support programs, volunteer driver programs, HCV chronic illness management education groups, and home support and volunteer visitor services for those on antiviral therapy. 

### 3.2. Determining the Benefits of Attendance

Patients described a decision process of determining or weighing the benefits of attending for care. Patients readily identified benefits in primary care related to attending for diagnosis, acute episodic care, and obtaining a specialist referral. Specialty care was circumscribed with benefits focused on issues surrounding treatment with a course of antiviral therapy or, in a few instances, management of advanced liver disease when treatment was not an option. 

Most patients did not report attendance for planned HCV monitoring in primary care. Those that reported planned care were being monitored for troublesome HCV symptoms or other conditions. For example, patients who reported regular laboratory monitoring of liver function in primary care relayed that these tests were required for methadone or HIV monitoring. Several explanations ensued regarding the absence of monitoring. 

Early in the disease course patients did not attend largely because of the absence of provider guidance on the need for monitoring following diagnosis. Patients relayed being advised to “live healthy,” eat a healthy diet, get exercise, and avoid alcohol. Provider participants confirmed this finding and said that youth were particularly at risk of not receiving any HCV followup. Providers explained that the lack of monitoring in primary care was linked to a knowledge gap and the paucity of detailed guidelines on long-term monitoring requirements in primary care. 

Patients made assumptions regarding the lack of followup advice. Some assumed that because of their addiction experiences they were not offered followup:

*I think that some of the physicians think because like I'm an ex drug addict, and I think they think that people that are drug addicts, I dunno, they figure that we're not going to pursue the treatment or you know we're not going to stick with it because we have addictions and everything and that's not the case … Like, maybe I wasn't told about it cuz maybe they saw it well like you know she has an addiction problem. *

Others assumed that followup was not advised because treatment was unavailable or they were ineligible. For example, a participant, diagnosed for four years, was surprised to learn about available treatments through the study. 

 Several patients said that they began to consider the need for monitoring when they learned more about HCV and observed HCV complications among their peers or experienced symptoms. In contrast, a few patients noted that, with increased knowledge, they were less likely to attend, “I knew as much as him, I did not see why I should go and use up that time.” 

Many patients reported that they did not attend for HCV care for years because they “felt fine” or had increased feelings of well-being after recovery from addictions. A few believed that the virus was “dormant” or not causing damage because they were taking care of themselves. However, others said they feared that the disease was a death sentence and thought that followup was futile: “it scared the crap out of me, I did not know anything about it. I thought that it was a disease that was gonna just progress so fast that, I actually thought I was gonna die from it, right?” 

With respect to treatment, many patients relayed that they sought treatment after they considered information from their peers, pamphlets, or the internet. Most patients said that they had to initiate the request for referral for treatment and be very assertive and persistent to obtain treatment:

*I've always known that it was my right to get healthcare even though I know certain nurses or doctors how they can treat you. You know I, I know that I have a right to healthcare just as much as anybody else you know whatever kind of coverage they have. I knew I had a right to it. So I, I never let that stop me. *

While some expressed a strong and urgent desire to undertake treatment, many spoke of weighing the risks and benefits. In preparation for therapy, fear of the risks from the required liver biopsy caused some to withdraw from care. One individual recalled how on three separate occasions he participated in the pretreatment workup until a liver biopsy was required:

*I've been doing this for many years now where I get to the point where I go in, I get an ultrasound done, I do my blood tests, I get my ultrasound done and then I'm supposed to get a biopsy on my liver and I usually, every time, I've chickened out and that's when I've relapsed and then I have to wait again. *

Providers relayed that the liver biopsy requirement can also affect future treatment opportunities:

* I have a case I remember, a patient is positive for hep C, has cancelled four times a liver biopsy, and that usually with our doctor we work for its strike three, strike four and that's it end of story. *

Other patients identified weighing the positive outcomes of antiviral therapy versus the difficult to tolerate side effects: “the point of treating something that's not bothering me at this point in time and make myself sick for the next six months, well, it does not make a lot of sense to me.”

For those who had experience with injection drug use, there were additional considerations:

* the other thing that scares me is being sick all the time because I, you know, being a, a junkie, when I was, or an addict or whatever, you know, you use that needle to make yourself better, you know, not to make yourself feel sick, right? *

Some decided to avoid experiencing treatment because the side effects would be “like going through withdrawal.” Others said that knowledge of rigours of treatment caused them to reject treatment until they could establish a more stable lifestyle and stronger support system. Others decided that the antiviral therapy success rates were too low and the personal costs too high, preferring to wait for newer improved therapies to emerge:

*some of the things I've seen people go through while on treatment and then come out at the end worse than they were when they started it, so that's one of the reasons I don't seek treatment until they get it right. *

Many expressed concerns about the mental and emotional impacts of antiviral therapy including the effects on their significant others. They were concerned that therapy would trigger a depression or volatile emotions that they often termed “ribo-rage.” Many said that they delayed HCV treatment because of what they heard from others who had experienced treatment, including opinions that regardless of the success rate they would not attempt treatment again. A participant who had prior experience with cancer chemotherapy recounted:

* I was very unwell … I went for chemotherapy for four years, I was in the palliative wing, I was very unwell and I survived that and I found this [HCV antiviral treatment] tougher. I found this tougher because it took away all of my fight, it took away because of the mental [impact], this was new territory for me. I've been near sick and dying before, I can do that—this was worse. *

A few said that they rejected treatment in the belief that “it is not a cure” or because they “felt pushed” by an emphasis on treatment at the specialist clinic. 

### 3.3. Competing Priorities

The competing priorities theme illuminates the place that HCV is given when patients have multiple and sometimes conflicting priorities in the other aspects of their life such as other health, work, or social needs. 

A number of patients described competing priorities of their work or family caregiver obligations. As one woman said, concern about HCV was supplanted by the needs of her sick husband:

* he was my number one thing to look after and my life and my health took a back seat to his because he was very, very sick and when he came home from the hospital he needed more aftercare … and he took up a lot of my time, you know and it, it just went by the wayside. *

Several patients said that they did not attend for care when they were actively using drugs. They described addictions as a “black hole” that absorbed them. A provider described youth who were addicted to crack as especially hard to engage because their lives were so chaotic. Related to drug use were disruptions in attendance for care on admittance to or release from prison. For example, treatment begun in prison would be interrupted on release until the person could access alternate resources in the community. Many said that prison release led to nonattendance for care because of other obstacles such as resumption of drug use or unstable living conditions. 

Social service providers identified that many of their clients with HCV have unstable income and living conditions, for various reasons, and those issues absorb their time and energy:

*I don't even know that some of the people that we work with who are homeless, who are street involved, whose lives are pretty chaotic, that they're even at a point where they feel that they have a choice, you know, because there are so many things that need to kind of get into place before the choice of treatment even enters their thinking about hep C. *

Patients spoke of needing to give time and priority to managing other coexisting conditions. One said “I got so much things wrong with me I, just like, oh, like it's my arthritis or whatever, like, I completely forgot I had hep C.” They also described not having the time to deal with HCV because the lack of an integrated approach required them to travel to a different provider for each condition, making their health management more complex. 

Many examples dealt with taking time out to realign life plans that patients described as necessary before accessing HCV treatment. It was a common belief that it was necessary to be recovered from addictions in order to access therapy, but there were other associated goals. For example, a young woman who used illicit drugs said that she was motivated to “get clean,” so she could access HCV treatment and be cured of the disease. She said her ultimate goal was to be healthy enough to “get her children back.” Other patient goals identified were to be healthy enough to start a family, protect family members or future sexual partners from the risk of transmission, be free from stigma, and prevent worse outcomes. 

For those with advanced liver disease, competing priorities were managing symptoms like fatigue and memory loss. Nonattendance resulted from not having enough energy to travel or simply forgetting about appointments. 

### 3.4. Knowledge Gaps

Gaps in patient and provider knowledge represent a prevalent theme in participant interviews. Knowledge gaps arose from a lack of knowledge, lack of understanding of information, misinterpretation of information, misinformation, and difficulty appraising information. 

Patients said that they obtained information through family, social and peer-networks, the internet, pamphlets, support groups, service providers, and community agencies such as drop-in centres. Information obtained focused on how HCV is acquired, prevention of transmission, and treatment with antiviral therapy. Patients described a lack of information that would help them sort out which of their symptoms were related to HCV, how often they should be monitored by a health care provider, what monitoring should involve, and how to most effectively self-manage their disease. This was especially relevant for those who did not or could not access antiviral therapy or for those who had not cleared their infection through therapy. Providers said that they were not aware of any standard recommendations for the type and frequency of monitoring for advancing liver disease in primary care, unlike specialty care where patient monitoring is individualized and based on specialist recommendations. 

Providers pointed out connections between the lack of monitoring and missed opportunities for patient education in primary care:

*we get a lot of calls about people asking if it's too late for treatment, if they've missed their chance for treatment and they're just people who maybe had hep C often for just a few years. So I think that also is a sign of these missed opportunities for conversations and … I think that's a sign that monitoring is not there because if people were going for monitoring they'd know, they would hopefully have a better sense of whether treatment is an option for them at this time. *

In addition, gaps in provider knowledge resulted in missed opportunities for patients to access services like testing and treatment. One provider said, “on the helpline, I receive calls from people from all over … and the first information they receive is from a doctor who … says that it cannot be treated.”

Participants relayed examples of omissions in diagnostic testing perceived to be related to inadequate provider knowledge. Two consecutive tests, a HCV antibody test followed by a HCV RNA test, are required to determine a chronic HCV infection, and yet many individuals have been told they had HCV, based solely on the antibody test, only to find out years later that this was not the case. 

A number of patients believed that gaps in provider knowledge resulted from the low profile assigned to HCV in training and education programs. For example, one patient recounted, “there's this street nurse that we have in [our small] town, she knows a bit about hepatitis but not that much because they did not give them a lot of training.” 

There were other missed opportunities for education when patients were diagnosed that impacted decisions to attend care. One woman said, “I found out in jail. They just gave me a pamphlet and I went back to my cell crying, cuz like, no education, I thought it was like, next thing to AIDS, yeah, no education.” 

Patients relayed that many individuals make the decision not to attend for antiviral therapy based on misinformation or the absence of information. For example, a reason for treatment deferral was a common belief within the drug using community that one had to be “clean” or off drugs and alcohol for at least six months prior to requesting treatment. A patient deferred treatment because he believed that he could not manage the 48-week treatment course. Had he known his HCV genotype required a 24-week treatment course, he would not have delayed. 

Many patients explained they frequently came across conflicting information requiring them to make decisions about which information to trust. Many privileged personal experience. For example, one patient said that the treatment information pamphlets did not correspond accurately with what he had seen friends endure. Nurse participants working in HCV treatment clinics said that they spend several sessions during the treatment preparation period assisting clients sort out “the myths and facts” and filling in knowledge gaps. They pointed out the dilemma of trying to establish a trusting relationship when patients disagree with or do not trust the accuracy of provider information. Providers identified the first few visits as critical to engaging patients and maintaining attendance for the duration of therapy. 

Sometimes patients felt overwhelmed by the complexity of information:

* I can't keep straight in my mind what tests I need to get from who and what blood work I need to get when. And what will happen to my body if I don't take these tests and get the blood works done. Also it would be good to know what the tests actually do and what they mean. *

Others had difficulties understanding HCV information such as the medical terms used by providers and were not comfortable exposing these difficulties. For example, many patients described their hepatitis as “dormant” or “resolved” but said that they were unsure what that meant. Others expressed problems wading through and understanding written information preferring to learn through other means:

*I really don't know nothing about hep C, I really like, it's um, it's just a word to me … because I don't have no information. I try to read it online, I read some but I couldn't understand it, like I needed to, for me to read something like that um, I couldn't understand but if somebody could explain it to me maybe I'd understand better. *

### 3.5. Access to Services

This theme encompasses the institutional (system) contributors that lead to nonattendance because services were either difficult to access or inaccessible. Issues include poverty, provider shortages, long wait times, few integrated or culturally appropriate services, and difficulty scheduling appointments. 

 Poverty or limited financial resources were an important contributor to nonattendance. Participants provided detailed examples of deferred or delayed care because patients did not have the financial resources for such things as drugs, transportation (which for specialist appointments often required travel to a large centre with high parking fees), phone calls, and childcare. Travel from rural/remote areas for specialist care was particularly difficult if the person needed to pay for a caregiver to accompany them or a hotel stay. 

 Provider shortages in primary and specialist care and long wait times restricted access resulting in nonattendance. Those in rural settings without family doctors said walk-in clinics were unavailable, leaving the hospital emergency the only option, which many would not access because of past negative experiences. A lack of local providers precipitated decisions not to access treatment, because treatment requires a local provider for managing urgent issues when patients live far from specialist clinics. Participants noted that there was a general shortage of specialists and supports such as social work, mental health, and addictions services. Where services existed, there were long wait times, which patients said deterred attendance. A provider from a large urban centre explained that “up to 50% of people referred to HCV specialists do not attend, and most of those are no shows.”

 In contrast, availability of integrated and culturally appropriate services (youth, Aboriginal, and immigrant) positively impacted attendance. Participants and providers said that patients were more likely to attend appointments if services were fully integrated, colocated, or organized as a one-stop-shop through a case manager. The types of services frequently mentioned for inclusion in integrated approaches included primary care, hepatitis specialty services, mental health, addictions, housing, and social services:

*they tried to do as much as they could for, for me in the appointment, right? And they had everybody in that one building so you know … the nurses and the psychiatrist and the doctors and, you know, um, yeah, that helped a lot, being able to just go to one spot. *

Providers confirmed that to improve attendance, patients need services based on the social determinants of health to “meet them where they are at.” This approach would ensure attention to such things as the contextual and cultural needs for youth, Aboriginal, and immigrant populations. 

 The time it takes to attend appointments and the frequency of appointments contributed to nonattendance. For example, the time spent in waiting rooms, sometimes for more than two hours, was a deterrent. The frequency of appointments required in preparation for a course of antiviral therapy complicated patients' life plans prompting nonattendance. For example, several patients spoke of making changes in their life plans that they thought necessary in anticipation of the six months to one year on therapy. They explained that these plans were disrupted by a long pre-treatment preparation time which included several months of waiting for and attending for various tests and consultations with multiple specialists prior to treatment initiation:

*… it's just been a long process and, and, and, you know, I wish when the doctor says you'd be starting it then that's when you start it, you don't get thrown all these other obstacles to get there which is, very, you know, cause you get all ready to do it and then you're like oh, now I have to do that and that's going to take three months, like that's wrong, right? You get yourself ready for it and then it's not happening. *

Providers confirmed that the time required for the pre-treatment evaluation is not widely communicated, even though it is essential to ensure the patient is fit to withstand the rigors of treatment. 

### 3.6. Restrictive Policies

Restrictive policies, the other institutional (system) level theme identified, impacted patients' ability to attend and engage with HCV care. Policies that impacted attendance ranged from local office policies to provincial system level requirements including requirements for a doctor's referral to a specialist; abstinence before consideration for treatment at some clinics; treatment eligibility criteria in many provinces; frequency of required clinic visits for treatment monitoring; and practices that restricted how or when services could be accessed. 

 Policies at the agency level that deterred attendance included limited calling times for making appointments, financial penalties, or service withdrawal for missed appointments and segregated appointment times or waiting rooms for those who used illicit drugs. Some patients pointed out that because of restrictive policies they dropped out of care and received only episodic care through drop-in clinics. 

 Many spoke of system level policies that affected their ability to access treatment. The requirement for doctor's referral to a hepatitis specialist was difficult to meet especially in areas with family physician shortages. In some provinces, treatment eligibility policies such as the requirement to demonstrate liver damage through repeated lab tests or liver biopsy acted as a deterrent. 

 Participants reported that those with addictions who needed to be enrolled in a methadone program prior to HCV therapy reported difficulties normalizing their life while complying with the program regulations because of daily reporting requirements, restricted dispensing times, and drug effects. Obtaining “carries” such as a three-day methadone supply allowed some freedom, but that privilege depended on the degree of adherence, length of time in the program, and the methadone prescriber:

*the methadone program the way it is set up in this province does not make somebody's life more livable because you gotta go to the pharmacy every couple of days, you gotta go to doctors appointments, you gotta go for mandated urine tests whether you're working or not, right, if you're out on a fishing boat you've gotta come in every three days to pick up your juice at the pharmacy, right? *

## 4. Discussion and Implications

This study explored the reasons for nonattendance for HCV care and identified factors affecting attendance at various points along the disease course: on diagnosis, postdiagnosis monitoring for disease progression, during preparation for and treatment with antiviral therapy once the disease had progressed, and in managing the disease when treatment was not an option. Analysis revealed nonattendance issues arise from the individual (patient or provider), patient/provider interactions, and institution (systemic) practices. The issues are contained within six interrelated themes: self-protection, competing priorities, determining the benefits, knowledge gaps, access to services, and restrictive policies. The findings from patients and HCV providers working in a variety of health and social service settings were congruent, confirming the work of Stewart who found concordance between patient, HCV specialist provider, and counsellor perspectives regarding HCV help-seeking and coping [33].

The study has several limitations. Participants were provided a small honorarium which may have influenced decisions to participate. In order to obtain a breadth and diversity of views, the participants were purposefully recruited, limiting the generalizeability of the findings. The results are based on participant recall which can be influenced by time and circumstances; for example, events in the present may be remembered with more clarity than distant events. However, the study's feedback loops during concurrent data collection and analysis provided opportunities to confirm, expand, or refute the points raised with subsequent participants. 

The issues identified in the self-protection theme extend the HCV stigma literature by describing the impact on care engagement when patients anticipate or experience being negatively judged or treated poorly. It confirms the association of HCV with illicit drug use and describes issues arising from the lack of provider HCV knowledge [27, 28, 34]. In accordance with Paterson et al., emergency room care was a context that elicited particularly strong negative patient responses and in this study led to nonattendance even at great personal health risk [27]. Drop-in clinics and integrated service centres were contexts where patients were more likely to have positive experiences. 

Determining the benefits of care, a recurrent theme throughout the disease course was of particular relevance on diagnosis as the absence of advice for monitoring or followup accounted for lengthy periods of nonattendance. Although there are specific guidelines for HCV assessment in primary care, they do not provide specifics for followup monitoring [35]. Closing the gap is crucial to improve secondary prevention of advanced liver disease and prevent premature deaths. As mortality studies have pointed out, interventions early in the course of the disease could prevent deaths from issues associated with HCV acquisition such as illicit drug use [36–38]. 

 While the theme knowledge gaps confirmed previously identified HCV knowledge deficits among primary care providers, it clearly identifies consequences for patients [39]. In accordance with the findings of Holman and Lorig, participants identified needs for accurate information and self-care education to cope with and adjust to their chronic illness [40]. The findings revealed that patients rely on knowledge gained outside the health system to make care decisions but were limited in their ability to access comprehensive information, fully understand medical terminology, or appraise the accuracy of information. For example, active drug use was cited as a reason for patients not accessing treatment and for providers not discussing treatment options with patients even though evidence supports treatment provision both from a clinical and cost-effectiveness stand point [41, 42]. In addition, decisions to undergo treatment are often made in the absence of a discussion with a health care provider, and the patient literature is silent on pretreatment workup requirements, which for some took up to a year and precipitated nonattendance. 

Competing priorities highlights the need to consider important aspects of lifestyle and other social determinants of health as well as disease specific aspects such as transmission and prevention, stages of illness, symptoms, prevention of progression, and options for antiviral therapy. This finding supports others who have identified the need for models of care that are patient-centered and integrate health and social care [9, 43]. Primary health care models that address complex health and social service needs similar to those of patients with chronic HCV have been shown to improve health outcomes [13, 44, 45]. 

The themes access to services and restrictive policies encompass multiple systemic obstacles faced by patients that impact care attendance such as lack of public transportation, provider shortages, long wait times, and the absence of culturally appropriate services. Restrictive policies were identified at single provider, agency, and provincial levels. These findings point to the need for system (policy) changes to improve service uptake such as: expanding specialist referral options to include self-referral and referral from allied health professionals; replicating approaches that improve access to specialists and reduce the need to travel to major centres by electronically linking medical specialists with generalist physicians in rural areas and nurse-led models of HCV prevention and care that include specialist consultation and have been shown to increase reach and access to care [18, 46, 47]. 

These results point to the need for clinical practice and system (policy) changes to increase engagement and retention in HCV care. Changes that would ensure patients are attended to in a respectful manner no matter what their background and that primary care providers have the knowledge and supports to provide patients with accurate and timely HCV information. Development and implementation of postdiagnosis monitoring protocols and mechanisms to improve knowledge dissemination in particular for nurses and physicians in primary care are recommended. 

## 5. Conclusion 

This is the first study to explore the reasons for nonattendance for HCV care throughout the disease course and validate the findings from multiple perspectives. It uncovered reasons for nonattendance, many of which are amenable through the provision of low barrier, nonjudgmental, and integrated services. It underscores the importance of provider and patient education that includes an emphasis on the psychological and sociocultural needs of the populations affected. This research can inform interventions, particularly in primary care, that are urgently required to engage and retain patients across the continuum of care, so that patients can realize the human and health system benefits of HCV care and emerging therapies that have the potential to cure 90–95% of those infected. 

## Figures and Tables

**Table 1:** Characteristics of patient participants (*n* = 55).

Variable	*N* (%)
Gender	
Male	30 (55)
Female	25 (45)
Age	
19–29	3 (5)
30–39	12 (22)
40–49	12 (22)
50+	28 (51)
Province	
BC	35 (64)
MB	1 (2)
ON	8 (15)
NS	10 (18)
NB	1 (2)
Education	
Elementary/some high school	17 (31)
Completed high school	12 (22)
Postsecondary	26 (47)
Source of income	
Disability/social assistance	42 (76)
Employed full or part time	13 (24)
Living arrangements	
Live alone	23 (42)
Live with family/others	32 (58)
Years since diagnosis	
<1	1 (2)
1–10	25 (45)
11–19	21 (38)
20+	8 (15)
